# Strangulated left paraduodenal hernia with jejunal necrosis managed in a field hospital: a case report

**DOI:** 10.1093/jscr/rjaf1099

**Published:** 2026-01-20

**Authors:** Khaled Alshawwa, Abed Dawoud, Amjad Abu– AlQumboz

**Affiliations:** General Surgery Department, Palestine Red Crescent Society Saraya Field Hospital, Gaza P860, Palestine; Faculty of Medicine, Al Azhar University of Gaza, Palestine; General Surgery Department, Palestine Red Crescent Society Saraya Field Hospital, Gaza P860, Palestine; Faculty of Medicine, Al Azhar University of Gaza, Palestine; General Surgery Department, Shifa Medical Complex, Gaza, Palestine; General Surgery Department, Palestine Red Crescent Society Saraya Field Hospital, Gaza P860, Palestine; Faculty of Medicine, Al Azhar University of Gaza, Palestine; General Surgery Department, Shifa Medical Complex, Gaza, Palestine

**Keywords:** paraduodenal hernia, internal hernia, strangulated hernia, closed-loop obstruction, emergency laparotomy, field hospital

## Abstract

Left paraduodenal hernia is the most common congenital internal hernia and a rare cause of acute small-bowel obstruction. Delay in diagnosis may lead to strangulation and bowel necrosis requiring urgent surgery. We report the case of a 30-year-old man presenting to a field hospital with sudden severe abdominal pain, vomiting, tachycardia, and generalized peritonism. Abdominal computed tomography demonstrated a cluster of jejunal loops in the left upper quadrant beneath the superior mesenteric vein, consistent with left paraduodenal hernia and closed-loop obstruction. Emergency midline laparotomy revealed a necrotic jejunal segment strangulated within the hernia sac. Approximately 60 cm of jejunum was resected, and a hand-sewn end-to-end anastomosis performed. A planned second-look laparotomy 48 hours later demonstrated complete bowel viability. Despite limited diagnostic resources in the conflict-zone field hospital, early operative intervention resulted in an uncomplicated recovery. This case underscores the need for high clinical suspicion and prompt surgery when internal hernia is suspected.

## Introduction

Paraduodenal hernias (PDH) are the most common congenital internal hernias, yet remain a rare cause of small-bowel obstruction. Left paraduodenal hernia (LPDH) occurs through Landzert’s fossa and typically presents as clustered jejunal loops in the left upper quadrant [1]. Early computed tomography (CT) descriptions by Suchato *et al.* characterized key radiologic features, including encapsulated bowel loops, mass effect on adjacent organs, and convergence of mesenteric vessels [1]. Later analyses emphasized that CT may suggest closed-loop obstruction even when overt ischemic signs are absent [2]. Clinically, PDH may remain asymptomatic for years before presenting acutely with obstruction and strangulation. In their systematic review, Schizas *et al.* reported that many patients require emergency surgery, and a substantial proportion require bowel resection when necrosis is present [3]. More recent reports highlight that extensive necrosis may occur when diagnosis is delayed [4].

## Case report

A 30-year-old male presented to the emergency department of a field hospital with severe generalized abdominal pain of a few hours’ duration, progressively worsening and unrelieved by any factor. He had two episodes of vomiting, no history of constipation, and no prior similar attacks. His past medical and surgical history was unremarkable.

On examination, he was tachycardic at 130–140 bpm, with blood pressure 115/70 mmHg and oxygen saturation 98%. His abdomen was distended with severe tenderness, maximal in the left upper and lower quadrants, accompanied by involuntary guarding. There were no abdominal wall changes or surgical scars. Rectal examination revealed an empty rectum.

**Figure 1:**
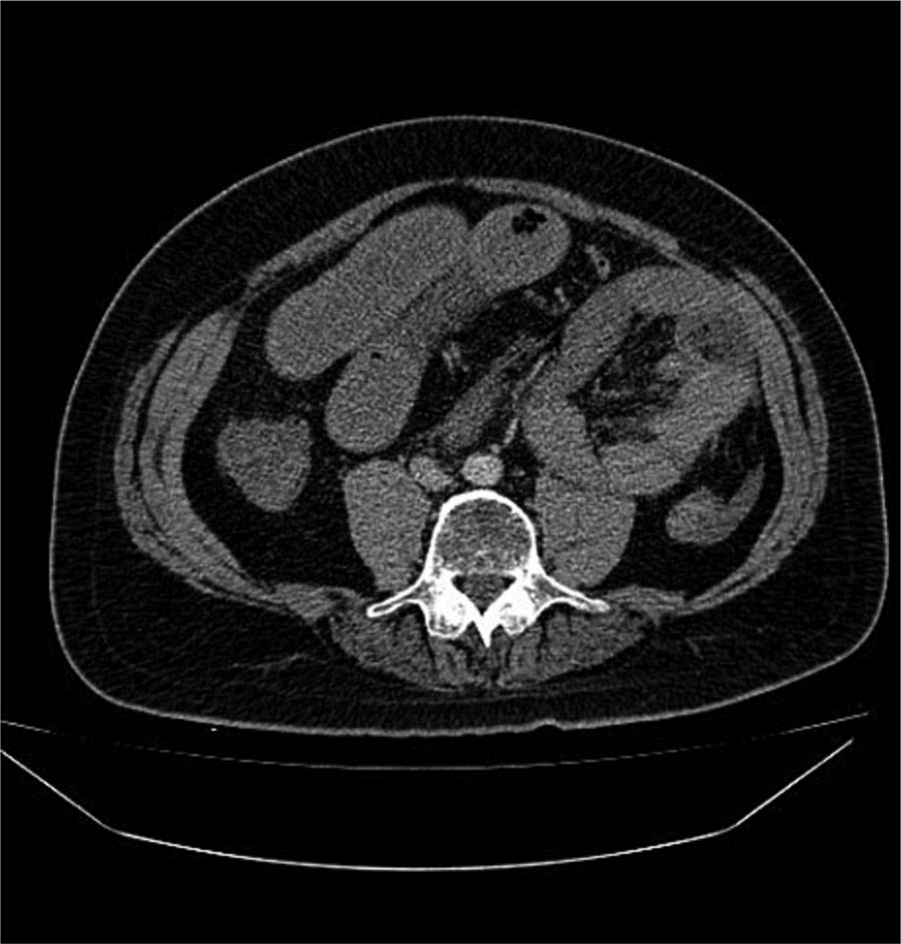
Contrast-enhanced axial CT abdomen: cluster of jejunal loops in the left upper quadrant forming a sac beneath the SMV, with proximal small-bowel dilatation consistent with closed-loop obstruction.

**Figure 2:**
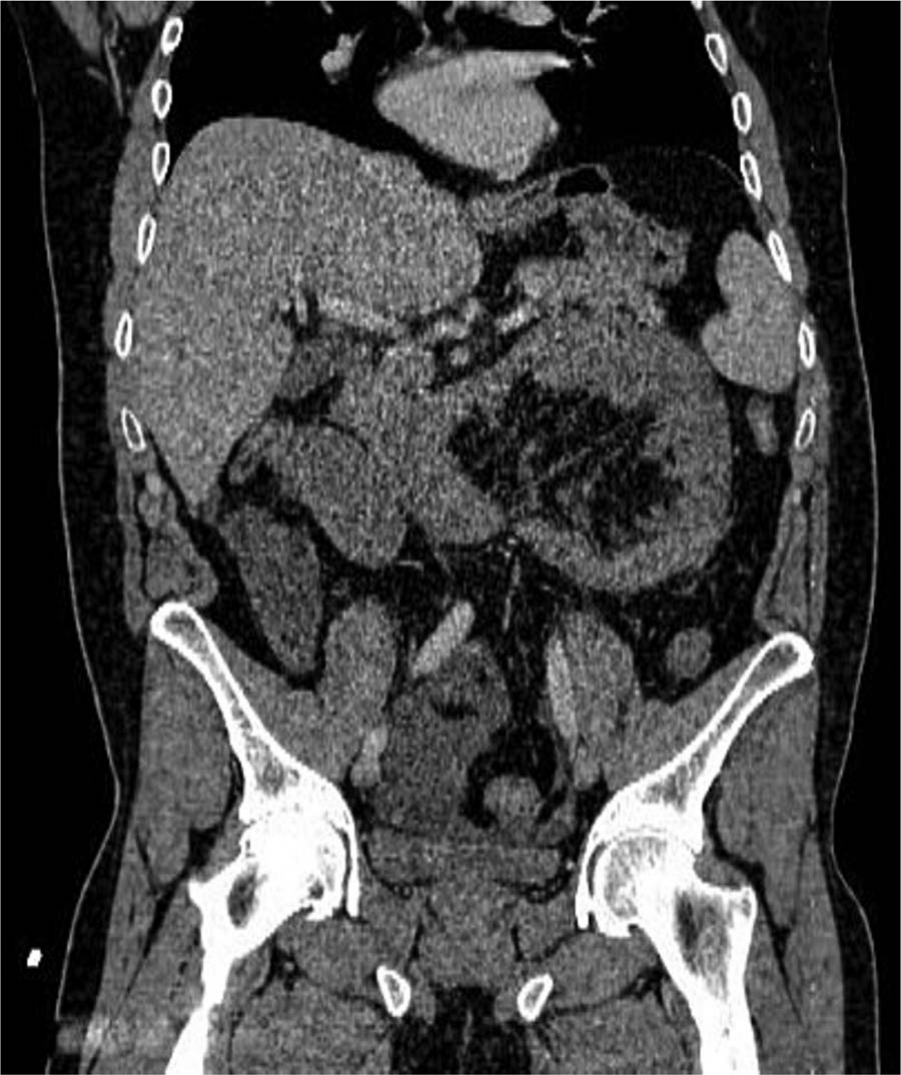
Coronal CT image showing clustered jejunal loops in the left abdomen with mass effect and convergence of mesenteric vessels toward the hernia orifice.

Abdominal radiography was non-contributory. Ultrasound demonstrated thickened small-bowel loops with inter-loop and minimal pelvic free fluid. Laboratory values showed WBC 20000/μl, Hb 15.4 g/dl, and platelets 417 × 10^9^/l. Contrast-enhanced CT, despite limited field-hospital imaging capability, revealed a sac of clustered jejunal loops in the left upper quadrant beneath the superior mesenteric artery, consistent with a LPDH causing closed-loop obstruction (Figs 1 and 2).

**Figure 3:**
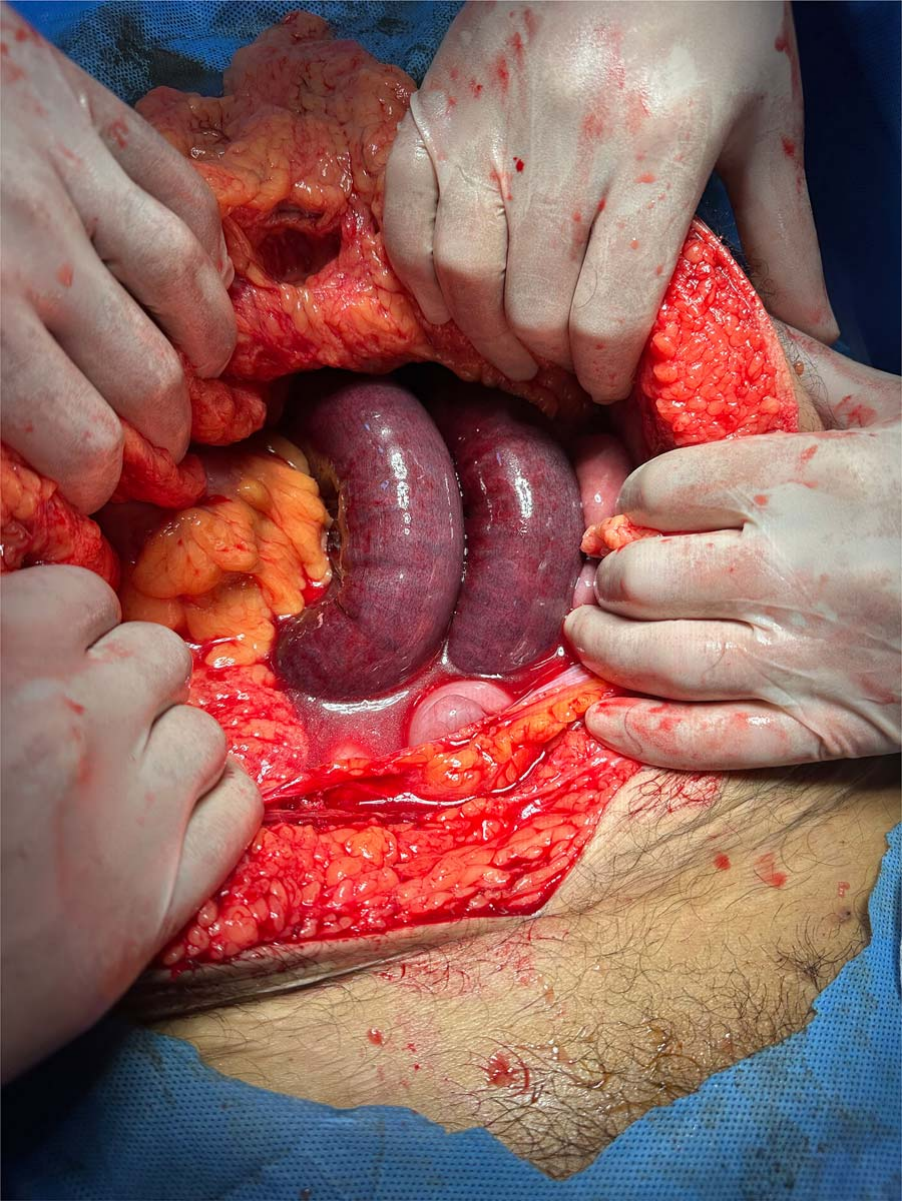
Intraoperative photograph demonstrating strangulated jejunal loops within the LPDH sac (note congestion and ischemic appearance).

**Figure 4:**
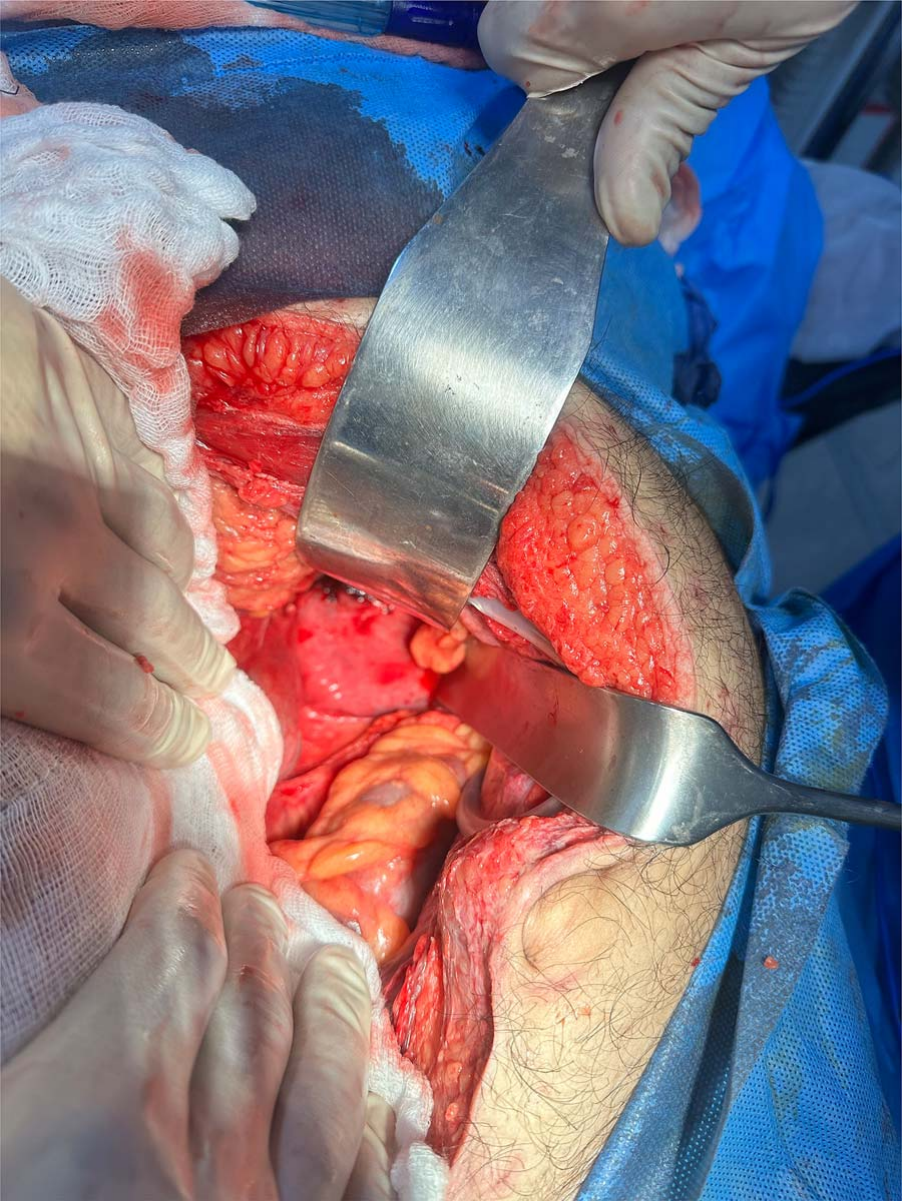
Shows left paraduodenal space after jejunal resection and excision of hernia sac*.*

The patient underwent urgent midline laparotomy. Moderate serous free fluid was present. A necrotic jejunal loop was found strangulated within a LPDH sac (Fig. 3). Other small-bowel loops were congested and erythematous but viable. Releasing the entrapped bowel did not result in improvement after a 5-minute observation period. Approximately 60 cm of jejunum, 15 cm distal to the duodenojejunal junction, was resected. A hand-sewn end-to-end anastomosis was performed. The hernia sac was excised with careful preservation of the superior mesenteric vein (SMV) (Fig. 4). A left-upper-quadrant drain was placed, and the abdomen was temporarily closed for a planned second-look.

During 48 hours in the intensive care unit, the patient’s condition improved steadily with normalization of vital signs and laboratory parameters. Second-look laparotomy confirmed a healthy anastomosis and viable bowel. The abdomen was closed definitively. Postoperative recovery was uncomplicated: the nasogastric tube was removed on day 1, oral intake advanced gradually, the drain was removed on day 2, and he was discharged home on postoperative day 3 from the second surgery. At 1-week follow-up, he remained well.

## Discussion

This case illustrates the classical progression of LPDH from occult congenital anomaly to sudden closed-loop obstruction with necrosis. In keeping with the early radiologic descriptions by Suchato *et al.* and Osadchy *et al.*, our patient’s CT demonstrated clustered loops beneath the SMA and convergence of mesenteric vessels, even though overt ischemia was not visualized [1, 2]. The discrepancy between imaging and operative findings is well documented, reinforcing the need for clinical judgment when strangulation is suspected.

Schizas *et al.* reported that many patients with PDH require emergency surgery and that bowel resection is common when presentation is delayed [3]. Sghaier *et al.* similarly described cases of extensive necrosis in congenital PDH [2]. Standard operative principles include reduction of herniated bowel, assessment of viability, resection of non-viable segments, and meticulous management of the hernia defect. Early reports emphasized caution due to the proximity of the inferior mesenteric vein and SMA branches [5]. Laparoscopic repair is increasingly reported for stable, non-strangulated PDH with favorable outcomes [6], but such resources were unavailable in the field hospital where this patient was managed.

Two lessons are highlighted by this case. First, in patients with acute small-bowel obstruction and no prior laparotomy, internal hernia must be strongly considered. Second, although CT is the diagnostic gold standard, operative intervention should not be delayed when peritoneal signs or systemic deterioration are present. In resource-limited or conflict-zone settings, where advanced imaging, laboratory tests, and minimally invasive surgery are unavailable, early surgical exploration is essential to minimize bowel loss and mortality.

## Conclusion

Left PDH is a rare but serious cause of acute small-bowel obstruction. In settings with limited diagnostic resources, early clinical suspicion and prompt operative intervention are crucial. Despite the austere field-hospital environment, timely surgery resulted in complete recovery.
